# Comparison of Two Wavefront Autorefractors: Binocular Open-Field versus Monocular Closed-Field

**DOI:** 10.1155/2020/8580471

**Published:** 2020-01-03

**Authors:** Gonzalo Carracedo, Carlos Carpena-Torres, Laura Batres, Maria Serramito, Anahí Gonzalez-Bergaz

**Affiliations:** ^1^Department of Optometry and Vision, Faculty of Optics and Optometry, Universidad Complutense de Madrid, Madrid, Spain; ^2^Ocupharm Diagnostics Research Group, Department of Biochemistry and Molecular Biology, Faculty of Optics and Optometry, Universidad Complutense de Madrid, Madrid, Spain

## Abstract

**Purpose:**

To evaluate the agreement and repeatability between a new commercially available binocular open-field wavefront autorefractor, as part of the Eye Refract system, and a monocular closed-field wavefront autorefractor (VX110).

**Methods:**

A cross-sectional, randomized, and single-masked study was performed. Ninety-nine eyes of 99 healthy participants (37.22 ± 18.04 years, range 8 to 69 years) were randomly analyzed. Three measurements with the Eye Refract and the VX110 were taken on three different days, under noncycloplegic conditions. Mean spherical equivalent (MSE), cylindrical vectors (J0 and J45), and binocular corrected distance visual acuity (BCDVA) were compared between both autorefractors. An intersession repeatability analysis was done considering the values of repeatability (*S*_*r*_) and its 95% limit (*r*).

**Results:**

The VX110 showed more negative values (*P* < 0.001) in terms of MSE in comparison with the Eye Refract (0.20 D). Regarding cylindrical vectors, J45 showed statistically significant differences (*P*=0.001) between both wavefront autorefractors, but they were not clinically relevant (<0.05 D). In BCDVA, there were no statistically significant differences (*P*=0.667) between both wavefront autorefractors. Additionally, the Eye Refract was more repeatable than the VX110 in terms of both MSE (*S*_*r*__EYE REFRACT_ = 0.21 D, *S*_*r*__VX110_ = 0.53 D) and J0 (*S*_*r*__EYE REFRACT_ = 0.12 D, *S*_*r*__VX110_ = 0.35 D).

**Conclusions:**

The Eye Refract provided enough accuracy and reliability to estimate refractive errors in different age groups, achieving better results than the VX110. Therefore, the Eye Refract proved to be a useful autorefractor to be incorporated into clinical practice.

## 1. Introduction

Subjective refraction is the gold standard method to assess the refractive error because it considers both optical and neural factors of visual perception. Nevertheless, autorefractors provide objective refraction as a starting point to facilitate subjective refraction.

Based on the current ways to measure objective refraction, autorefractors can be classified as monocular or binocular, closed- or open-field, and traditional or wavefront-based [1–4]. Binocular or monocular indicates if the binocularity is present or not during the measurement, while closed- or open-field alludes to whether the image of fixation is virtual or real.

Besides autorefractors that use traditional methods to measure objective refraction, wavefront autorefractors have been developed to become a usual device in clinical practice, especially during the last decade [5]. The main limitation of wavefront autorefractors is an overestimation of myopia or underestimation of hyperopia [6–8]. This issue is also important for traditional autorefractors, both monocular closed-field [3, 9–12] and binocular closed-field [9, 13]. Despite this limitation, some models of wavefront autorefractors demonstrated a good agreement with subjective refraction in terms of spherical and cylindrical refractive errors [2, 14–18].

Binocular open-field traditional autorefractors were developed to avoid myopia that a monocular closed-field environment may be generated without cycloplegia [19]. This idea is supported by different studies that showed similar results between these autorefractors and subjective refraction in terms of spherical refractive error [1, 4, 20–23].

In the case of wavefront autorefractors, all the commercially available models are monocular closed-field, except a new binocular open-field wavefront autorefractor, as part of the Eye Refract system (Luneau Technology; Chartres, France). The clinical implications and limitations of the Eye Refract are still unknown.

For this reason, the purpose of the current study was to evaluate the agreement and repeatability between this new binocular open-field wavefront autorefractor (Eye Refract) and a monocular closed-field wavefront autorefractor (VX110). Both systems are commercially distributed by Luneau Technology (Chartres, France).

## 2. Methods

### 2.1. Design of the Study

A cross-sectional, randomized, and single-masked study was performed. The study was conducted in compliance with good clinical practice guidelines and institutional review board regulations and following the tenets of the Declaration of Helsinki, revised and actualized in 2013 [24]. All trials were performed in the University Clinic of Optometry of the Universidad Complutense de Madrid (Madrid, Spain). All participants were voluntarily included in the study after signing a written informed consent, where the procedure of all the trials and the purpose of the study were explained. Participants were free to leave the study at any time.

Three measurements of the objective refraction with two wavefront autorefractors (Eye Refract and VX110) were taken. One of the autorefractors was used randomly in first place and the other in second place. All the measurements were performed on three different days (one measurement with each wavefront autorefractor per day) in the morning by the same optometrist, under noncycloplegic conditions. All visits of each participant were done during a maximum period of two weeks based on their availability. Refractive parameters (MSE, J0, and J45) and binocular corrected distance visual acuity (BCDVA) were compared between both wavefront autorefractors.

### 2.2. Sample

Ninety-nine eyes of 99 healthy participants (37.22 ± 18.04 years, range 8 to 69 years) were evaluated, considering one eye per participant randomly. The recruitment was made to obtain the most heterogeneous sample as possible concerning the age of participants, trying to involve the same number of participants in each decade of life. The participants were classified into four groups (total, teenagers, adults, and presbyopes), whose demographic characteristics are detailed in Table 1.

Inclusion criteria were as follows: aged between 7 and 69 years and understanding and signing the informed consent (by the legal tutors, in case of participants under 18 years). Exclusion criteria were amblyopia, strabismus, or other ocular dysfunction affecting the binocular autorefraction, the presence of any ocular disease, surgery or traumatism, and the use of systemic or ocular drugs that could affect the results.

### 2.3. Eye Refract System

The Eye Refract system (Luneau Technology; Chartres, France) is a binocular open-field aberrometer combined with a phoropter. The Eye Refract incorporates two Hartmann–Shack sensors that perform objective refraction in both eyes at the same time. The wavefront metric used for the objective refraction determination is based on the principle of equivalent quadratic, using the method of paraxial curvature matching proposed by Thibos et al. [25] This method considers the high-order aberrations analysis up to 4^th^ order, using the Zernike coefficients *C*_2_^0^ and *C*_4_^0^ for MSE determination, *C*_2_^2^ and *C*_4_^2^ for J0 determination, and *C*^2_−2_^ and *C*_4_^−2^ for J45 determination The Eye Refract measures the wavefront under physiological pupil conditions, and it recalculates the refractive parameters for 3 mm. If pupil size is equal to or less than 3 mm, the Eye Refract provides the values for its exact size. The Hartmann–Shack sensors use a near-infrared light of 800 nm, and the pitch of the microlens array is 0.1 mm.

Since the Eye Refract is an aberrometer combined with a phoropter, it provides both objective and subjective refraction. The results of its efficacy to perform aberrometry-based subjective refraction in comparison with conventional subjective refraction were already published [26]. In the current manuscript, we only report on the results of objective refraction.

Following the manufacturer instructions, subjects were instructed to put their chin and forehead on the chinrest and to look ahead to a test of fixation on the digital screen set at 4 m distance. Then, binocular wavefront aberrometry was measured.

### 2.4. VX110 System

The VX110 system (Luneau Technology; Chartres, France) is a multidiagnostic platform that incorporates an aberrometer [2]. The VX110 has a Hartmann–Shack sensor that performs a monocular and closed-field objective refraction. The wavefront metric used for the objective refraction determination is also based on the principle of equivalent quadratic, using the method of paraxial curvature matching proposed by Thibos et al. [25] All the refractive variables were considered for a pupil size of 3 mm. If pupil size is equal to or less than 3 mm, the VX110 provides the values for its exact size. The Hartmann–Shack sensor also uses a near-infrared light of 800 nm, and it measures 1500 points for a pupil diameter of 7 mm.

Following the manufacturer instructions, subjects were instructed to put their chin and forehead on the chinrest and to look ahead to a virtual image of fixation at infinity. Then, monocular wavefront aberrometry was performed in each eye consecutively.

### 2.5. Refractive Parameters

Refractive parameters were analyzed in terms of mean spherical equivalent (MSE) and vertical and oblique cylindrical vectors (J0 and J45) with the method proposed by Thibos et al. [27]. The following expressions were used to calculate MSE, J0, and J45:

  MSE = sphere + cylinder/2
  J0 = −(cylinder/2) × cos (2 *x* axis)
  J45 = −(cylinder/2) × sin (2 *x* axis)

### 2.6. Visual Acuity Measurement

Binocular corrected distance visual acuity (BCDVA) was immediately measured after finishing each objective refraction. BCDVA was assessed through the oculars of the Eye Refract, in the case of the objective refraction with this system, and with trial frame, in the case of the objective refraction with the VX110. The high-contrast (100%) ETDRS chart of the digital screen set at 4 meters of distance was used to measure the BCDVA.

### 2.7. Statistical Analysis

Statistical analysis was performed using the SPSS Statistics 23 software (IBM, Chicago, Illinois, USA). Sample size calculations were performed with statistical software Granmo 6.0 (Institut Municipal d'Investigació Mèdica, Barcelona, Spain). A statistical significance of 95% was established (*P* < 0.05). Results are shown as mean ± standard deviation.

The normality of the variables (MSE, J0, J45, and BCDVA) was assessed using the Shapiro–Wilk test. A statistical comparison between the values of both wavefront autorefractors inside each group (total, teenagers, adults, presbyopes) was done. Once the normal distribution of all the variables was confirmed, Student's *t* test for paired samples was chosen for this statistical comparison. Additionally, a Bland–Altman plot analysis was done to assess the agreement between both wavefront autorefractors [28].

An intersession repeatability analysis was done considering the following variables: mean difference between sessions (bias), its standard deviation (SD), repeatability (*S*_*r*_), and its 95% limit (*r*). *S*_*r*_ is defined as the square root of the mean square within-subject standard deviation. *r* is mathematically defined as 2.77 × *S*_*r,*_ and it represents the limit value within which 95% of measurements should be [29]. The one-way analysis of variance (ANOVA) for paired samples with Bonferroni correction was done to assess the statistical differences between sessions.

## 3. Results

Table 2 summarizes the mean values of the different variables under study (MSE, J0, J45, and BCDVA) for both wavefront autorefractors and their statistical comparison.

**Figure 1:**
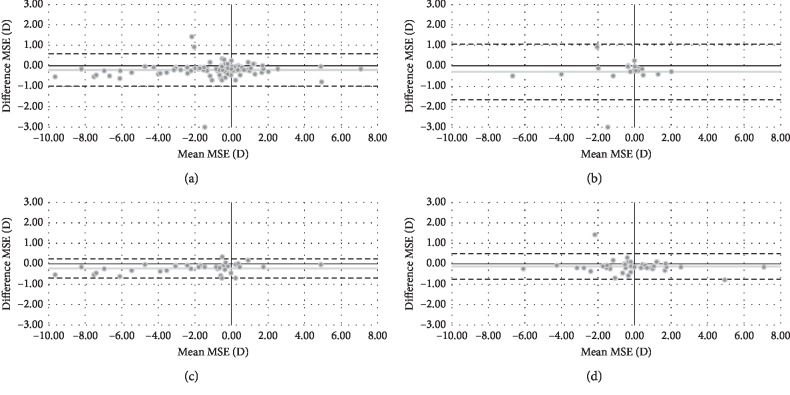
Bland–Altman plot describing the agreement between mean spherical equivalent (MSE) of both wavefront autorefractors for total group (a), teenagers (b), adults (c), and presbyopes (d). The middle line shows the mean difference (VX110-Eye Refract), and the two dashed side lines show the 95% limits of agreement.

In relation to MSE, the VX110 showed more negative values (*P* < 0.05) in comparison with the Eye Refract for all groups, except teenagers.  Figure 1 shows the Bland–Altman plot for both objective refractions in terms of MSE. Mean difference (VX110-Eye Refract) and limits of agreement [upper, lower] were −0.20 [0.59, −0.99] D for total group, −0.30 [1.06, −1.66] D for teenagers, −0.24 [0.22, −0.70] D for adults, and −0.13 [0.50, −0.76] D for presbyopes.

**Figure 2:**
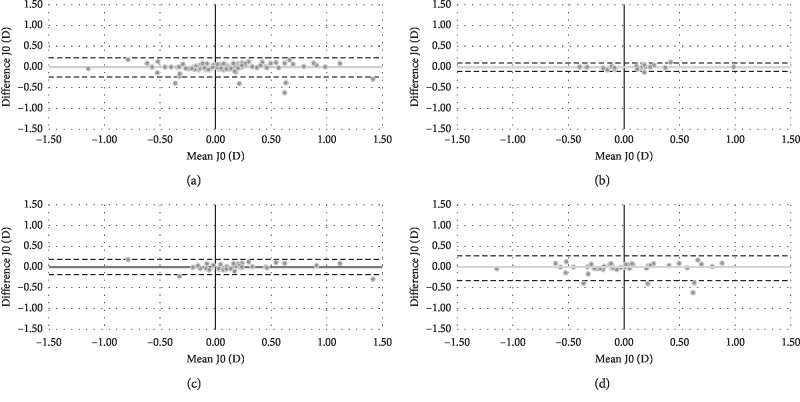
Bland–Altman plot describing the agreement between J0 of both wavefront autorefractors for total group (a), teenagers (b), adults (c), and presbyopes (d). The middle line shows the mean difference (VX110-Eye Refract), and the two dashed side lines show the 95% limits of agreement.

In relation to J0, there were no statistically significant differences (*P* ≥ 0.05) between both wavefront autorefractors.  Figure 2 shows the Bland–Altman plot for both objective refractions in terms of J0. Mean difference (VX110-Eye Refract) and limits of agreement [upper, lower] were −0.02 [0.22, −0.24] D for total group, 0.00 [0.10, −0.10] D for teenagers, 0.00 [0.18, −0.18] D for adults, and −0.03 [0.27, −0.33] D for presbyopes.

**Figure 3:**
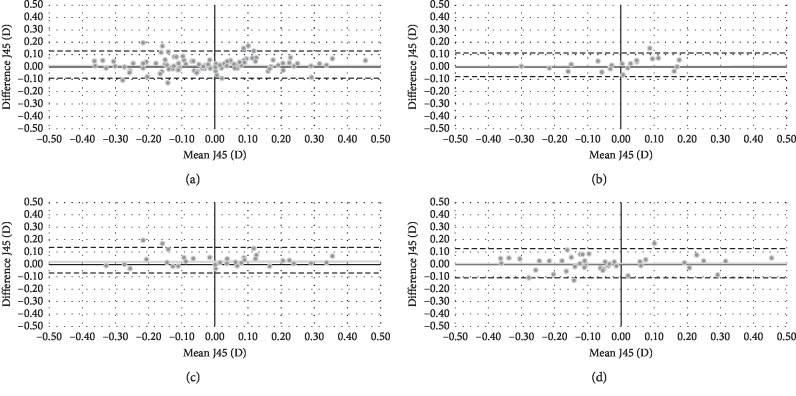
Bland–Altman plot describing the agreement between J45 of both wavefront autorefractors for total group (a), teenagers (b), adults (c), and presbyopes (d). The middle line shows the mean difference (VX110-Eye Refract), and the two dashed side lines show the 95% limits of agreement.

In relation to J45, there were statistically significant differences (*P* < 0.05) between both wavefront autorefractors for total group and adults.  Figure 3 shows the Bland–Altman plot for both objective refractions in terms of J45. Mean difference (VX110-Eye Refract) and limits of agreement [upper, lower] were 0.02 [0.13, −0.09] D for total group, 0.01 [0.11, −0.09] D for teenagers, 0.04 [0.14, −0.06] D for adults, and 0.00 [0.12, −0.12] D for presbyopes.

**Figure 4:**
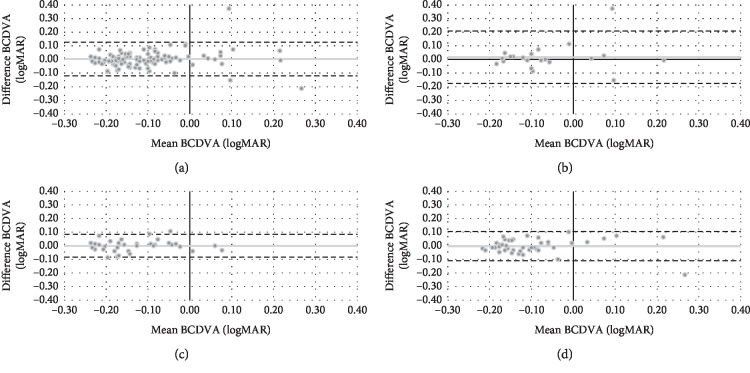
Bland–Altman plot describing the agreement between binocular corrected distance visual acuity (BCDVA) of both wavefront autorefractors for total group (a), teenagers (b), adults (c), and presbyopes (d). The middle line shows the mean difference (VX110-Eye Refract), and the two dashed side lines show the 95% limits of agreement.

In relation to BCDVA, there were no statistically significant differences (*P* ≥ 0.05) between both wavefront autorefractors.  Figure 4 shows the Bland–Altman plot for both objective refractions in terms of J45. Mean difference (VX110-Eye Refract) and limits of agreement [upper, lower] were 0.00 [0.12, −0.12] D for total group, 0.02 [0.21, −0.17] D for teenagers, 0.00 [0.08, −0.08] D for adults, and 0.00 [0.10, −0.10] D for presbyopes.

Tables 3–5 summarize the intersession repeatability analyses of the refractive variables (MSE, J0, and J45, respectively) for both wavefront autorefractors and the statistical comparison between sessions.

The intersession repeatability analysis of MSE showed no statistically significant differences (*P* ≥ 0.05) between sessions with both wavefront autorefractors. The Eye Refract was more repeatable than the VX110 for all the groups, especially for teenagers (*S*_*r*__EYE REFRACT_ = 0.14 D, *S*_*r*__VX110_ = 0.92 D). In total group, the values of *S*_*r*_ were 0.21 D with the Eye Refract and 0.53 D with the VX110.

The intersession repeatability analysis of J0 only showed statistically significant differences (*P*=0.033) between session 1 and session 3 for adults with the Eye Refract. The Eye Refract was also more repeatable than the VX110 for all the groups. In total group, the values of *S*_*r*_ were 0.12 D with the Eye Refract and 0.35 D with the VX110.

The intersession repeatability analysis of J45 showed statistically significant differences (*P* < 0.05) between session 1 and session 3 for total group and presbyopes with the VX110. The VX110 was more repeatable than the Eye Refract for all the groups, especially for presbyopes (*S*_*r*__EYE REFRACT_ = 0.15 D, *S*_*r*__VX110_ = 0.08 D). In the total group, the values of *S*_*r*_ were 0.11 D with the Eye Refract and 0.06 D with the VX110.

## 4. Discussion

The current study is the first to evaluate the performance of a binocular open-field autorefractor (Eye Refract) based on wavefront analysis. The performance of the Eye Refract was compared with the VX110, a monocular closed-field autorefractor. The results showed that the objective refraction performed by the VX110 was more negative in terms of MSE than the Eye Refract for all groups, except teenagers. Additionally, the Eye refract was more repeatable in terms of both MSE and J0 than the VX110 for all groups, but the VX110 was more repeatable in terms of J45.

Overestimation of myopia or underestimation of hyperopia has been the main limitation of wavefront autorefractors since they appeared on the market until nowadays [6–8], but this issue also affects traditional autorefractors, both monocular closed-field [3, 9–12] and binocular open-field [9, 13]. More negative values in the sphere are associated with the stimulation of the accommodation during the measurement process. For the total group of the current study, the VX110 showed more negative values in terms of MSE in comparison with the Eye Refract (0.20 D) (see Table 2). These differences could be considered clinically relevant since they were close to 0.25 D, values which could be affecting vision [30]. Additionally, the Bland–Altman plot for both objective refractions (Figure 1) showed more negative values with the VX110 in most of the participants. This trend was continued in all age groups. Also, teenagers were the only group that did not have statistical differences in MSE between both wavefront autorefractors (Table 2). Nevertheless, the difference for teenagers was maximum (0.30 D) in comparison with the rest of the groups. This maximum difference without statistical significance was associated with a single participant who obtained more negative values (around 3.00 D) with the VX110 (Figure 1(b)).

As explained above, the overestimation of myopia with the VX110 could be associated with higher stimulation of accommodation [6–8]. However, it seems that this problem could also affect to open-field autorefractors [21], which would not explain the differences between both wavefront autorefractors. Besides, this overestimation with the VX110 only affected adults and presbyopes who are supposed to be the groups of age with lower accommodation.

Considering that accommodative response was not evaluated, it is necessary to explore other theories that could explain the differences found in MSE between both wavefront autorefractors. The results of Elsner et al. [31] and Teel et al. [32] suggested that the differences in the sphere could be associated with the infrared light of the wavefront sensors reflecting off in external retinal layers. However, this theory would not explain the differences in the sphere between both wavefront refractors since they use the same near-infrared light of 800 nm. The fact that the Eye Refract measured the refraction with a test of fixation at 4 m and the VX110 with a virtual image of fixation at infinity would not support the differences in MSE either since the Eye Refract uses a fogging with positive lenses before measuring objective refraction.

On the other hand, it is known that convergence is accompanied by accommodation [33]. By this, it could be thought that the differences in MSE between both wavefront autorefractors were related to the fact that both measurements were performed under binocular and monocular conditions. Despite this, it would be necessary for an additional study about the influence of the vergence and accommodation in both wavefront autorefractors to confirm this theory.

The authors of the current study reported the values of MSE obtained with conventional subjective refraction [26]. The values of MSE were −0.86 D for the total group, −0.63 D for teenagers, −2.08 D for adults, and +0.25 D for presbyopes. Comparing these values with the current study (Table 2), it can be observed that the Eye Refract only showed an overestimation of myopia superior to 0.25 D for presbyopes (*P*=0.021). However, the VX110 showed an overestimation of myopia superior to 0.25 D for adults and presbyopes (*P* < 0.001) [26]. This overestimation of myopia found with the VX110 contrast with the results of Gordon-Shaag et al. [2]. They found no differences in MSE with the same wavefront autorefractor in comparison with the subjective refraction performed by a single optometrist. These differences between both studies could be explained considering that they only performed one refraction by the same optometrist who could have its prescription criteria. The results of the current study concerning the VX110 also contrast with other authors that did not report differences in the spherical refraction between the newest wavefront autorefractors and subjective refraction [2, 15–18]. Nevertheless, some authors reported more negative sphere values with different wavefront aberrometers in comparison with subjective refraction during the last decade [8, 32, 34–36], coinciding with the results obtained with the VX110 in the current study. No studies assessing the performance of commercial open-field wavefront autorefractors were found in the scientific literature.

In terms of intersession repeatability of MSE, the Eye Refract was more repeatable than the VX110 for all the groups and sessions (Table 3), but both autorefractors were very similar for adults. The major benefit of the Eye Refract was for teenagers since its value of *S*_*r*_ (0.14 D) was 6.5 times lower than the *S*_*r*_ of the VX110 (0.92 D). However, this affirmation should be carefully interpreted because the sample of teenagers was inferior to the rest of the groups (Table 1).

Other studies analyzed the intersession repeatability of MSE with different wavefront autorefractors. In adults, several authors reported values of *r* between 0.28 D and 0.53 D [2, 16, 23, 37, 38], which agrees with the adults of the current study (Table 3). All these wavefront autorefractors keep their differences within a range of ±0.25 D. No studies assessing the intersession repeatability with wavefront autorefractors in other age groups were found in the scientific literature.

Astigmatism is a parameter that wavefront autorefractors offer properly [7, 8, 14–18]. Only a few studies reported differences when comparing cylinder between wavefront autorefractors and subjective refraction [2, 6], but these differences were approximate of 0.25 D, which is a value well tolerated by the human eye [39]. In the current study, despite there were statistically significant differences in cylindrical component J45 between both wavefront autorefractors for some groups, they could not be considered clinically relevant since the mean differences only reached a maximum value of 0.04 D (Table 2). Therefore, it could be affirmed that both wavefront autorefractors have the accuracy to properly determine astigmatism for all age groups since they did not show higher differences in comparison with conventional subjective refraction (*P* ≥ 0.05) [26].

The intersession repeatability of astigmatism showed that the Eye Refract was more repeatable than the VX110 (Table 4) for all age groups, while the VX110 was more repeatable than the Eye Refract in terms of J45 (Table 5). In J45, since the differences between both wavefront autorefractors in terms of *r* were inferior to 0.25 D (except for presbyopes) and that the magnitude of J45 obtained with both wavefront autorefractors was substantially lower than MSE and J0 (Table 2), the improvement in J45 repeatability with the VX110 was not considered clinically relevant. In adults, other studies reported similar values of *r* in terms of J0 and J45 with different wavefront autorefractors [2, 16, 23, 37, 38].

In terms of BCDVA, there were no statistical differences between both wavefront autorefractors for all age groups (Table 2). In the case of the VX110, it is logical to think that BCDVA is not affected although it seems to overestimate myopia in comparison with the Eye Refract. This is due to the capacity of the lens to compensate for a negative overcorrection, especially in teenagers and adults. Presbyopes would have more difficulty to accommodate, but their negative overcorrection was not higher to 0.75 D in any participant (Figure 1(d)). In comparison with conventional subjective refraction [26], both wavefront autorefractors showed a statistically significant deterioration (*P* < 0.05) of BCDVA for all the groups, except with the Eye Refract for teenagers (*P*=0.126). In total group, this deterioration was only 0.02 logMAR (1 letter), which is not considered clinically relevant [40].

The current study had some limitations that could be improved upon in future studies. Visual acuity was measured under unmasked conditions and using trial frame with the VX110 and through the oculars with the Eye Refract. A study of Ohlendorf et al. [41] showed that phoropter could induce more negative spherical values than trial frame. On the other hand, all the objective refractions should have been performed under cycloplegic conditions to prove that the VX110 measurement is not influencing the accommodation stimulus because it is thought that a closed-field environment could overestimate myopia [19]. Also, it is necessary to check if these autorefractors could replace conventional subjective refraction since the objective refraction of the Eye Refract presented similar efficacy to subjective refraction [26]. Finally, more studies would be necessary for understanding the efficacy, applications, and limitations of open-field binocular wavefront autorefractors.

## 5. Conclusions

In conclusion, the VX110 showed more negative values in terms of MSE than the Eye Refract. Besides, the Eye Refract was more repeatable in terms of both MSE and J0 than the VX110. Both wavefront autorefractors showed similar results in terms of J45 and BCDVA.

The Eye Refract provided enough accuracy and reliability to estimate refractive errors in different age groups, achieving better results than the VX110. Therefore, the Eye Refract proved to be a useful autorefractor to be incorporated into clinical practice.

## Figures and Tables

**Table 1:** Demographic characteristics of the participants in the study.

Groups	Number of participants	Age (years)	Age range (years)	Gender (male/female)
Teenagers	21	13.05 ± 4.31	8 to 19	9/12
Adults	33	29.89 ± 5.71	22 to 39	8/25
Presbyopes	45	55.00 ± 8.14	40 to 69	18/27
Total	99	37.22 ± 18.04	8 to 69	35/64

**Table 2:** Mean values of mean spherical equivalent (MSE), cylindrical components (J0 and J45), and binocular corrected distance visual acuity (BCDVA) obtained with both binocular open-field (eye refract) and monocular closed-field (VX110) autorefractors.

Parameter	Wavefront autorefractor	Total group (*n* = 99)	Teenagers (*n* = 21)	Adults (*n* = 35)	Presbyopes (*n* = 43)
MSE (D)	Eye refract	−0.85 ± 2.65	−0.50 ± 1.86	−2.19 ± 3.17	−0.03 ± 2.16
	VX110	−1.05 ± 2.69	−0.80 ± 1.94	−2.43 ± 3.25	−0.16 ± 2.11
	*P* value	<0.001^*∗*^	0.065	<0.001^*∗*^	0.002^*∗*^

J0 (D)	Eye refract	0.10 ± 0.41	0.09 ± 0.31	0.19 ± 0.42	0.03 ± 0.43
	VX110	0.08 ± 0.40	0.09 ± 0.32	0.19 ± 0.40	0.00 ± 0.42
	*P* value	0.241	0.622	0.669	0.161

J45 (D)	Eye refract	−0.01 ± 0.30	−0.01 ± 0.12	−0.01 ± 0.18	0.00 ± 0.41
	VX110	0.01 ± 0.31	0.00 ± 0.14	0.03 ± 0.18	0.00 ± 0.42
	*P* value	0.001^*∗*^	0.168	0.004^*∗*^	0.095

BCDVA (logMAR)	Eye refract	−0.10 ± 0.10	−0.07 ± 0.11	−0.14 ± 0.08	−0.09 ± 0.11
	VX110	−0.10 ± 0.11	−0.05 ± 0.13	−0.14 ± 0.08	−0.09 ± 0.11
	*P* value	0.667	0.419	0.716	0.978

The results are expressed as mean ± SD. The statistical comparison was done between both autorefractors inside each group. ^*∗*^*P* < 0.05, Student's *t* test for paired samples.

**Table 3:** Intersession repeatability of mean spherical equivalent (MSE) obtained with both binocular open-field (eye refract) and monocular closed-field (VX110) autorefractors in terms of mean difference between sessions (bias), its standard deviation (SD), repeatability (*S*_*r*_), and its 95% limit (*r*).

Wavefront autorefractor	Group	MSE	Session 1-session 2	Session 1–session 3	Session 2-session 3	Repeatability [95% limit] (D)
Eye refract	Total	Bias ± SD (D)	−0.02 ± 0.33	0.00 ± 0.28	0.02 ± 0.29	0.21 [0.59]
		*P* value	1.000	1.000	1.000	
	Teenagers	Bias ± SD (D)	−0.08 ± 0.16	−0.10 ± 0.24	−0.02 ± 0.19	0.14 [0.40]
		*P* value	0.102	0.188	1.000	
	Adults	Bias ± SD (D)	−0.02 ± 0.21	−0.02 ± 0.23	0.00 ± 0.20	0.15 [0.41]
		*P* value	1.000	1.000	1.000	
	Presbyopes	Bias ± SD (D)	0.01 ± 0.45	0.06 ± 0.32	0.04 ± 0.37	0.27 [0.75]
		*P* value	1.000	0.692	1.000	

VX110	Total	Bias ± SD (D)	−0.07 ± 0.40	−0.05 ± 0.87	0.02 ± 0.88	0.53 [1.47]
		*P* value	0.325	1.000	1.000	
	Teenagers	Bias ± SD (D)	−0.18 ± 0.77	0.18 ± 1.52	0.36 ± 1.49	0.92 [2.55]
		*P* value	0.906	1.000	0.843	
	Adults	Bias ± SD (D)	−0.06 ± 0.28	−0.09 ± 0.22	−0.04 ± 0.23	0.18 [0.50]
		*P* value	0.760	0.071	1.000	
	Presbyopes	Bias ± SD (D)	−0.02 ± 0.17	−0.12 ± 0.77	−0.10 ± 0.76	0.44 [1.23]
		*P* value	1.000	0.933	1.000	

The statistical comparison was done between sessions. ^*∗*^*P* < 0.05, one-way ANOVA for paired samples with Bonferroni correction.

**Table 4:** Intersession repeatability of vertical cylindrical vector (J0) obtained with both binocular open-field (eye refract) and monocular closed-field (VX110) autorefractors in terms of mean difference between sessions (bias), its standard deviation (SD), repeatability (*S*_r_), and its 95% limit (*r*).

Wavefront autorefractor	Group	J0	Session 1-session 2	Session 1–session 3	Session 2-session 3	Repeatability [95% limit] (D)
Eye refract	Total	Bias ± SD (D)	−0.04 ± 0.19	−0.03 ± 0.12	0.02 ± 0.19	0.12 [0.34]
		*P* value	0.077	0.059	1.000	
	Teenagers	Bias ± SD (D)	−0.04 ± 0.10	−0.03 ± 0.10	0.01 ± 0.09	0.07 [0.20]
		*P* value	0.276	0.767	1.000	
	Adults	Bias ± SD (D)	−0.03 ± 0.09	−0.04 ± 0.09	−0.01 ± 0.10	0.06 [0.18]
		*P*-value	0.171	0.033^*∗*^	1.000	
	Presbyopes	Bias ± SD (D)	−0.06 ± 0.27	−0.02 ± 0.14	0.04 ± 0.26	0.16 [0.46]
		*P* value	0.478	1.000	0.982	

VX110	Total	Bias ± SD (D)	0.02 ± 0.22	−0.01 ± 0.10	−0.04 ± 0.20	0.35 [0.98]
		*P* value	0.909	0.355	0.196	
	Teenagers	Bias ± SD (D)	−0.01 ± 0.09	−0.02 ± 0.08	−0.02 ± 0.08	0.26 [0.73]
		*P* value	1.000	0.588	1.000	
	Adults	Bias ± SD (D)	0.02 ± 0.15	0.00 ± 0.10	−0.02 ± 0.13	0.33 [0.92]
		*P* value	1.000	1.000	1.000	
	Presbyopes	Bias ± SD (D)	0.04 ± 0.29	−0.02 ± 0.10	−0.06 ± 0.27	0.40 [1.11]
		*P* value	1.000	0.502	0.458	

**Table 5:** Intersession repeatability of oblique cylindrical vector (J45) obtained with both binocular open-field (Eye Refract) and monocular closed-field (VX110) autorefractors in terms of mean difference between sessions (bias), its standard deviation (SD), repeatability (*S*_r_), and its 95% limit (*r*).

Wavefront autorefractor	Group	J45	Session 1-session 2	Session 1–session 3	Session 2-session 3	Repeatability [95% limit] (D)
Eye refract	Total	Bias ± SD (D)	−0.02 ± 0.20	−0.01 ± 0.15	0.01 ± 0.11	0.11 [0.30]
		*P* value	0.812	1.000	1.000	
	Teenagers	Bias ± SD (D)	0.00 ± 0.06	0.02 ± 0.10	0.02 ± 0.10	0.06 [0.18]
		*P* value	1.000	1.000	1.000	
	Adults	Bias ± SD (D)	0.00 ± 0.10	0.00 ± 0.09	0.00 ± 0.07	0.06 [0.18]
		*P* value	1.000	1.000	1.000	
	Presbyopes	Bias ± SD (D)	−0.05 ± 0.28	−0.04 ± 0.20	0.01 ± 0.13	0.15 [0.41]
		*P* value	0.671	0.565	1.000	

VX110	Total	Bias ± SD (D)	0.02 ± 0.10	0.02 ± 0.07	0.00 ± 0.10	0.06 [0.18]
		*P* value	0.215	0.031^*∗*^	1.000	
	Teenagers	Bias ± SD (D)	0.02 ± 0.07	0.01 ± 0.06	0.00 ± 0.07	0.04 [0.12]
		*P* value	0.831	0.796	1.000	
	Adults	Bias ± SD (D)	−0.01 ± 0.07	0.00 ± 0.06	0.01 ± 0.07	0.04 [0.12]
		*P* value	1.000	1.000	1.000	
	Presbyopes	Bias ± SD (D)	0.04 ± 0.12	0.03 ± 0.08	−0.01 ± 0.12	0.08 [0.77]
		*P* value	0.160	0.038^*∗*^	1.000	

The statistical comparison was done between sessions. ^*∗*^*P* < 0.05, one-way ANOVA for paired samples with Bonferroni correction.

## Data Availability

The data used to support the findings of this study are available from the corresponding author upon request.

## References

[1] Sheppard A. L., Davies L. N. (2010). Clinical evaluation of the grand seiko auto ref/keratometer WAM-5500. *Ophthalmic and Physiological Optics*.

[2] Gordon-Shaag A., Pinero D. P., Kahloun C. (2018). Validation of refraction and anterior segment parameters by a new multi-diagnostic platform (VX120). *Journal of Optometry*.

[3] Paudel N., Adhikari S., Thakur A., Shrestha B., Loughman J. (2019). Clinical accuracy of the nidek ARK-1 autorefractor. *Optometry and Vision Science*.

[4] Wosik J., Patrzykont M., Pniewski J. (2019). Comparison of refractive error measurements by three different models of autorefractors and subjective refraction in young adults. *Journal of the Optical Society of America A*.

[5] Applegate R., Atchison D., Bradley A. (2014). Wavefront refraction and correction. *Optometry and Vision Science*.

[6] Nissman S. A., Tractenberg R. E., Saba C. M., Douglas J. C., Lustbader J. M. (2006). Accuracy, repeatability, and clinical application of spherocylindrical automated refraction using time-based wavefront aberrometry measurements. *Ophthalmology*.

[7] Reinstein D. Z., Archer T. J., Couch D. (2006). Accuracy of the WASCA aberrometer refraction compared to manifest refraction in myopia. *Journal of Refractive Surgery*.

[8] Zhu X., Dai J., Chu R., Lu Y., Zhou X., Wang L. (2009). Accuracy of WASCA aberrometer refraction compared to manifest refraction in Chinese adult myopes. *Journal of Refractive Surgery*.

[9] Choong Y.-F., Chen A.-H., Goh P.-P. (2006). A comparison of autorefraction and subjective refraction with and without cycloplegia in primary school children. *American Journal of Ophthalmology*.

[10] Kinge B., Midelfart A., Jacobsen G. (1996). Clinical evaluation of the allergan humphrey 500 autorefractor and the nidek AR-1000 autorefractor. *British Journal of Ophthalmology*.

[11] Gwiazda J., Weber C. (2004). Comparison of spherical equivalent refraction and astigmatism measured with three different models of autorefractors. *Optometry and Vision Science*.

[12] Pesudovs K., Weisinger H. S. (2004). A comparison of autorefractor performance. *Optometry and Vision Science*.

[13] Queirós A., González-Méijome J., Jorge J. (2008). Influence of fogging lenses and cycloplegia on open-field automatic refraction. *Ophthalmic and Physiological Optics*.

[14] Salmon T. O., West R. W., Gasser W., Kenmore A. T. (2003). Measurement of refractive errors in young myopes using the COAS Shack-Hartmann aberrometer. *Optometry and Vision Science*.

[15] Cooper J., Citek K., Feldman J. M. (2011). Comparison of refractive error measurements in adults with Z-view aberrometer, Humphrey autorefractor, and subjective refraction. *Optometry-Journal of the American Optometric Association*.

[16] Shneor E., Millodot M., Avraham O., Amar S., Gordon-Shaag A. (2012). Clinical evaluation of the L80 autorefractometer. *Clinical and Experimental Optometry*.

[17] McGinnigle S., Naroo S. A., Eperjesi F. (2014). Evaluation of the auto-refraction function of the Nidek OPD-Scan III. *Clinical and Experimental Optometry*.

[18] Lebow K. A., Campbell C. E. (2014). A comparison of a traditional and wavefront autorefraction. *Optometry and Vision Science*.

[19] Mallen E. A. H., Gilmartin B., Wolffsohn J. S., Tsujimura S.-I. (2015). Clinical evaluation of the Shin-Nippon SRW-5000 autorefractor in adults: an update. *Ophthalmic and Physiological Optics*.

[20] Mallen E., Wolffsohn J. S., Gilmartin B., Tsujimura S. (2001). Clinical evaluation of the Shin-Nippon SRW-5000 autorefractor in adults. *Ophthalmic and Physiological Optics*.

[21] Chat S., Edwards M. H. (2001). Clinical evaluation of the Shin-Nippon SRW-5000 autorefractor in children. *Ophthalmic and Physiological Optics*.

[22] Davies L. N., Mallen E. A. H., Wolffsohn J. S., Gilmartin A. B. (2003). Clinical evaluation of the shin-nippon NVision-K 5001/grand seiko WR-5100K autorefractor. *Optometry and Vision Science*.

[23] Cleary G., Spalton D. J., Patel P. M., Lin P.-F., Marshall J. (2009). Diagnostic accuracy and variability of autorefraction by the tracey visual function analyzer and the Shin-Nippon NVision-K 5001 in relation to subjective refraction. *Ophthalmic and Physiological Optics*.

[24] World Medical Association Declaration of Helsinki (2013). Ethical principles for medical research involving human subjects. *JAMA*.

[25] Thibos L. N., Hong X., Bradley A., Applegate R. A. (2004). Accuracy and precision of objective refraction from wavefront aberrations. *Journal of Vision*.

[26] Carracedo G., Carpena-Torres C., Serramito M., Batres-Valderas L., Gonzalez-Bergaz A. (2018). Comparison between aberrometry-based binocular refraction and subjective refraction. *Translational Vision Science & Technology*.

[27] Thibos L. N., Wheeler W., Horner D. (1997). Power vectors: an application of fourier analysis to the description and statistical analysis of refractive error. *Optometry and Vision Science*.

[28] Bland J. M., Altman D. G. (1999). Measuring agreement in method comparison studies. *Statistical Methods in Medical Research*.

[29] McAlinden C., Khadka J., Pesudovs K. (2015). Precision (repeatability and reproducibility) studies and sample-size calculation. *Journal of Cataract & Refractive Surgery*.

[30] Atchison D. A., Schmid K. L., Edwards K. P., Muller S. M., Robotham J. (2001). The effect of under and over refractive correction on visual performance and spectacle lens acceptance. *Ophthalmic and Physiological Optics*.

[31] Elsner A. E., Burns S. A., Weiter J. J., Delori F. C. (1996). Infrared imaging of sub-retinal structures in the human ocular fundus. *Vision Research*.

[32] Teel D. F. W., Jacobs R. J., Copland J., Neal D. R., Thibos L. N. (2014). Differences between wavefront and subjective refraction for infrared light. *Optometry and Vision Science*.

[33] Judge S. J. (1996). How is binocularity maintained during convergence and divergence?. *Eye*.

[34] Kilintari M., Pallikaris A., Tsiklis N., Ginis H. S. (2010). Evaluation of image quality metrics for the prediction of subjective best focus. *Optometry and Vision Science*.

[35] Jinabhai A., O’Donnell C., Radhakrishnan H. (2010). A comparison between subjective refraction and aberrometry-derived refraction in keratoconus patients and control subjects. *Current Eye Research*.

[36] Hastings G. D., Marsack J. D., Nguyen L. C., Cheng H., Applegate R. A. (2017). Is an objective refraction optimised using the visual Strehl ratio better than a subjective refraction?. *Ophthalmic and Physiological Optics*.

[37] Dobos M. J., Twa M. D., Bullimore M. A. (2009). An evaluation of the bausch & lomb zywave aberrometer. *Clinical and Experimental Optometry*.

[38] Otero C., Vilaseca M., Arjona M., Martínez-Roda J. A., Pujol J. (2015). Repeatability of aberrometric measurements with a new instrument for vision analysis based on adaptive optics. *Journal of Refractive Surgery*.

[39] Villegas E. A., Alcón E., Artal P. (2014). Minimum amount of astigmatism that should be corrected. *Journal of Cataract & Refractive Surgery*.

[40] Bailey I. L., Bullimore M. A., Raasch T. W., Taylor H. R. (1991). Clinical grading and the effects of scaling. *Investigative Ophthalmology & Visual Science*.

[41] Ohlendorf A., Leube A., Wahl S. (2016). Steps towards smarter solutions in optometry and ophthalmology-inter-device agreement of subjective methods to assess the refractive errors of the eye. *Healthcare*.

